# Crime scene and body alterations caused by arthropods: implications in death investigation

**DOI:** 10.1007/s00414-018-1883-8

**Published:** 2018-06-24

**Authors:** A. Viero, M. Montisci, G. Pelletti, S. Vanin

**Affiliations:** 1 Legal Medicine and Toxicology, University-Hospital of Padova, Via Falloppio, 50, 35121 Padova, Italy; 2 Department of Medical and Surgical Sciences, Unit of Legal Medicine, University of Bologna, Bologna, Italy; 3 Department of Biological Sciences, School of Applied Sciences, University of Huddersfield, Huddersfield, UK

**Keywords:** Forensic entomology, Forensic pathology, Crime scene alteration, Body artefacts

## Abstract

The activity of arthropods on corpses has been largely investigated, since they can produce information to reconstruct the peri-mortem events. However, the feeding/movement activity of insects around the crime scene, among the clothes and on the body, can also cause some alterations that can lead to wrong reconstruction and misinterpretations. This article summarises all the post-mortem arthropods artefacts related to the scene (i.e. fly artefacts and floor stripes) and the body (i.e. skin and other soft tissue alterations, bone alterations and hair alterations) that can mislead the forensic pathologist, discussing macroscopic and microscopic findings derived from forensic casework and from experimental laboratory studies, in order to provide a useful instrument to avoid misinterpretations and evaluation errors. Finally, some procedural notes for the documentation and the interpretation of findings are proposed.

## Introduction

Arthropods, including insects, arachnids, scorpions and crustaceans, are the largest and most numerous zoological taxon on Earth. Arthropods are present in a wide variety of locations commonly used by humans, and they have been reported also from crime scenes.

During the last three decades, the utility of forensic entomology in death investigation has been largely documented in Europe, America, Australia, and Asia by several case studies [1] and experimental works that represent the references for the discipline.

It is worth mentioning that such cases and experiments show the potentiality of this discipline in the forensic pathology investigation field not only in post-mortem interval (PMI) estimation but also in drug detection, cadaver transfer and victim identification [2]. Therefore, the arthropods found at a crime scene and on a victim’s body can assist many types of forensic investigation [1, 3]. In particular, they can provide information about time since death, season of death, primary crime scene, movement or concealment of the remains following death, specific sites of trauma on the body, use of drugs and neglect of children [4], elders [5] or non-autonomous people [6] and victim’s identification when the body is removed from the initial decomposition site [2].

Despite being highly informative, arthropods have the potential to strongly alter and modify the crime scene and the victim’s body.

Therefore, given the complexity of crime scene investigations and the necessity to respond to the six W questions (i.e. What, Where, How, When, Who and Why) and to understand the dynamics of violent crimes, the forensic entomological knowledge is of utmost importance, and the forensic pathologists should be aware of the damage that arthropods can cause, together with the useful information they can provide.

The aim of this work is to analyse the scientific literature dealing with all the post-mortem artefacts, caused by arthropods, of the scene and the body in order to provide forensic pathologists with a useful instrument to avoid misinterpretations and evaluation errors.

In fact, a summary overview of these lesions in a single paper has never been done until now and this article can be considered as a consultative tool for forensic pathologists and crime scene investigators, during a crime scene investigation and the autopsy.

## Materials and methods

### Review of the literature

A literature search in the electronic databases Pubmed, Scopus and Web of Science was conducted using a combination of free text protocols (i.e. “insects”, “flies”, “artefacts”, “post-mortem”, “forensic”, “crime scene”, “body”, “blood”, “stains”) individually combined through the Boolean operator “AND”. At the same time, filters such as full-text, publication date from January 2000 to September 2016, and English language were activated resulting in more than 100 articles, which were submitted to the following criteria of inclusion (one at least):

- description of post-mortem artefacts in death scene;
- presentation of post-mortem cases investigated through (at least) an autopsy with or without histology analyses;
- critical discussion on issues related to the identification or misinterpretation of post-mortem artefacts.

Moreover, entomological books and forensic entomology and pathology manuals [7–12], which include chapters dedicated to the description of post-mortem artefacts in death scene, were also included.

Subsequently, the selected papers were analysed in full text together with the chapters of the selected books and manuals.

## Results and discussion

Forty-one papers dealing with post-mortem artefacts due to arthropods that fulfilled the inclusion criteria were included in the investigation. Among these, 7 were case reports [13–19], 3 case series [6, 20, 21], 19 original articles [22–40], 4 were technical notes [41–44] and 8 were reviews [1, 10, 45–50].

As previously mentioned, insects from a crime scene and/or a cadaver can provide useful information; however, their feeding and locomotor activity through the crime scene, among the clothes and on/in the body, can also cause some alterations resulting, if wrongly interpreted, to misinterpretations and wrong investigative reconstructions.

The analysis of the literature shows that several authors described the alterations and modifications of the crime scene and the post-mortem injuries caused by different animals: vertebrates and invertebrates. The following paragraphs describe and discuss the alterations of the crime scene, clothes and body generated by the feeding and locomotor activity of invertebrates, saprophagous arthropods, associated with the cadaver decomposition. These alterations can be grouped as “crime scene entomological alterations” (i.e. fly specks, floor and wall stripes) and “entomological body artefacts” (i.e. skin lesions and other soft tissue alterations, bone alterations, hair alterations).

### Crime scene entomological alterations

Bloodstain pattern analysis (BPA) has a key role during crime scene investigation, providing crucial information about the physical events that led to the bloodstain deposition [49]. The information obtained by BPA can be used for the reconstruction of the manner of death and the evaluation of other primary issues related to the death, such as the victim’s and perpetrator’s position and movement before and after committing the crime and the assessment of witnesses’ statements [48].

Since bloodstains are not static and can be altered after formation, in particular during the liquid phase, some alterations after deposition should be considered as part of the BPA (i.e. transfer, contact and drip patterns, expired blood or high velocity spatter), and they could allow the reconstruction of the criminal acts [19, 48, 51]. Problems can arise when other modifications, such as those produced by the insect activity, alter the bloodstains and consequently their interpretation. These alterations are commonly defined fly artefacts (FAs), when produced by the action of blowflies, or, more generally, insect stains [52].

Morphological alterations of bloodstain pattern due to insect activity are described in several entomological textbooks [7–9], but, until now, few real cases have been reported in the forensic literature [22, 37, 47, 53, 54]. This discrepancy could be due to a lack of consciousness regarding the ability of insects to alter the death scene, producing artefacts that could be misinterpreted as bloodstains produced during the crime.

### Fly artefacts

FAs were described for the first time by Lassaigne in 1856 [55], who defined them as small stains transferred from a blood source to a blood-free surface by the action of blowflies. FAs are produced by the tarsi of flies, that come in contact with a blood source, so that blood-soaked tarsi can get in touch with any surface [7]. Contamination can also be the result of the digestive process, because of the nature of the fly feeding, consisting in a first extra-oral digestion due to the excretion of some enzymes, followed then by the ingestion of the food in a liquid state. Blow flies ingest blood using a sponging mouthparts and then regurgitate the ingested food by partially expelling it as a bubble and then sucking it back in. In addition, flies can also defecate material derived by the digestions of blood or decomposing fluids on the crime scene [8].

The differentiation between FAs and bloodstains and the confounding factors affecting their shapes and characteristics have been widely discussed in forensic literature [47]. Benecke et al. [22, 56] reported three cases in which differential diagnoses between bloodstain patterns and FAs were crucial in determining the manner of death. The same authors described some features of FAs (e.g. stains that have a tail-to-body ratio greater than 1, stains with a tadpole/sperm type structure, stains with a sperm cell-type structure that do not end in a small dot. stains without a distinguishable tail and body, stains that do not participate in directional consistency with other stains that suggest a point of convergence at a point of origin) that can be useful for distinguishing the FAs from the bloodstains. However, these features are still the matter of debate [53, 57].

The same authors [22] also provided general rules to be followed during crime scene investigation, including the documentation of fly activity and of the range of stains. They reported as well how to compare the FAs observed on the crime scene with other known fly artefact patterns. They concluded that the presence of spots far from sources of blood is not suggestive of a bloodstain because blowflies are able to deposit FAs also in room in which blood is not present [25]. When dealing with a group of spots, the comparison among the droplets pertaining to the same pattern can be a useful method for the distinction between bloodstains and FAs. Laboratory analyses performed on different species of blowflies showed that FAs are characterised by a wide range of size, shape, reflectance, lack of congruent directionality or consistent colouration and a distribution that appears evenly spaced [37]. One of the indicators used to distinguish FAs from bloodstain is that artefacts have tails going in directions that were contrary to the majority of drops [25]. Blood ingested by flies is not completely digested before defecation and may even pass through a fly digestive system without any degradation. For this reason, faecal matter resembles to blood both from a biomolecular and chemical point of view: these artefacts contain enough human blood to give positive results with haem-based presumptive tests [25, 42, 50]. As a result, chemical and chemo-fluorescent presumptive blood tests are unable to distinguish blood from FAs so they have to be used with caution/attention before making any hypothesis. Immunological confirmatory tests (which detect the presence of haemoglobin) have been demonstrated to be more useful, being able to distinguish between 3-day-old artefacts from blood, and less reliable in the differentiation between 2-week-old artefacts from biological fluid [41]. However, some differences were observed between the manner of deposition, and consequently the appearance, of FAs, even between different species. The surface can also influence the deposition and, consequently, the shape of FAs. As an example, the so-called “carpet avoidance behaviour” implies that on a few fly stains are observed in crime scene where blood pool or stains occurred primarily in porous surfaces, such as carpets [43]. Moreover, porous surfaces can modify the deposited morphology of artefacts or bloodstain, so that investigators have to be aware of potential contamination by FAs when looking at drops on clothing, carpet or other porous and irregular surfaces [37].

Even if there are not universal rules that can be used to differentiate FAs from bloodstains, Table 1 summarises some characteristics that could suggest the origin of a suspicious spot or group of spots.

**Table 1:** Features that can suggest the nature of suspect blood spots found during a crime scene investigation. In case of a group of spots, differentiation between bloodstains and FA relies on the comparison between the suspect spot(s) and other spots belonging to the same pattern. In case of a single spot, the differentiation is based on the assessment of some particular features that could be the result of fly activity. In both cases, there are not universal rules that can be followed for the distinction between bloodstain and FAs, solely based on macroscopic examination of the spots

Multiple spots		Single spot	
Bloodstain Pattern [22, 37]	Fly artefacts [22, 25, 37, 48]	Bloodstain pattern [37]	Fly artefacts [22, 26, 37]
Same colour and/or shade of colour	Different colour/colour shade	Spines/scalloped edges	No distinguishable tail and body
All spots of elliptical/circular shape	Different/irregular shape	Linear and/or multiple tails	Curved or non-linear tail
Relationship shape/impact angle	Tails with opposite directions	Body length > 20 mm	Tail longer than body with irregular morphology
Consistent directionality	Random directionality		Textured surface
Radiating pattern	Absence of a point of convergence		Cratered appearance
Linear/curvilinear orientation			Evidence of flow

In addition to macroscopic observations, immunological and molecular tests have to be applied for the correct identification of suspect spots [35].

On the other side, flyspecks can be a further source of human DNA useful for victim identification if the body is removed from the crime scene before the investigators’ arrival. From this point of view, flies can be considered as a vehicle for the spreading of the victims DNA. Human DNA can be extracted from FAs in sufficient quantities to provide a full profile of the donor [27–30, 34, 41]. The amount of DNA that can be extracted, and so the number of FAs required to generate a forensic relevant profile, depends on the biological material ingested by the fly (i.e. blood, semen, saliva) [30]. DNA can be extracted from FAs derived from blood and semen for a period of at least 2 years, and from saliva for 2 months after deposition [27, 28]. As a result, artefacts can be a valuable source of DNA for investigators in cases where the victim’s body was removed and/or the offender attempted to clean up any biological evidence due to the ability to be sampled a long time after deposition and far from the crime scene. This last event can be also considered as a potential source of contamination, and particular attention has to be paid in case of an exogenous DNA profile is found in a crime scene.

### Insect stains

**Fig. 1:**
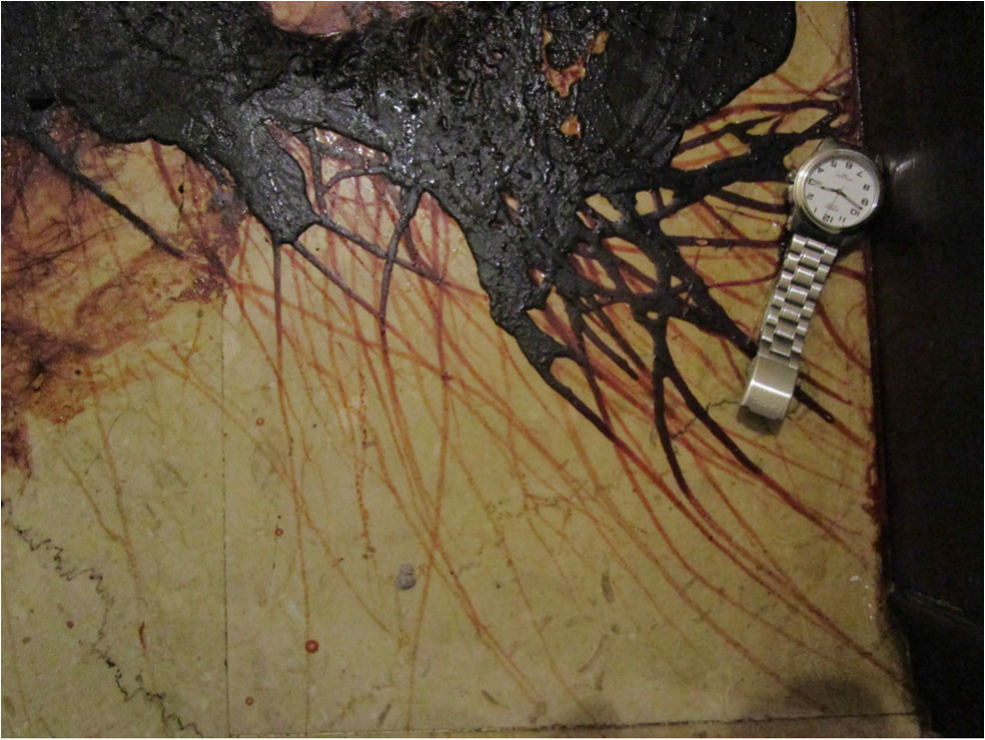
Floor stripes due to the wandering of maggots from the body to protected places where pupation occurs. If migrating from a surface soaked by blood or putrefactive liquid, maggots leave a linear wipe pattern, resulting in a series of trails produced by their typical crawling motion (Photo by S. Vanin)

**Fig. 2:**
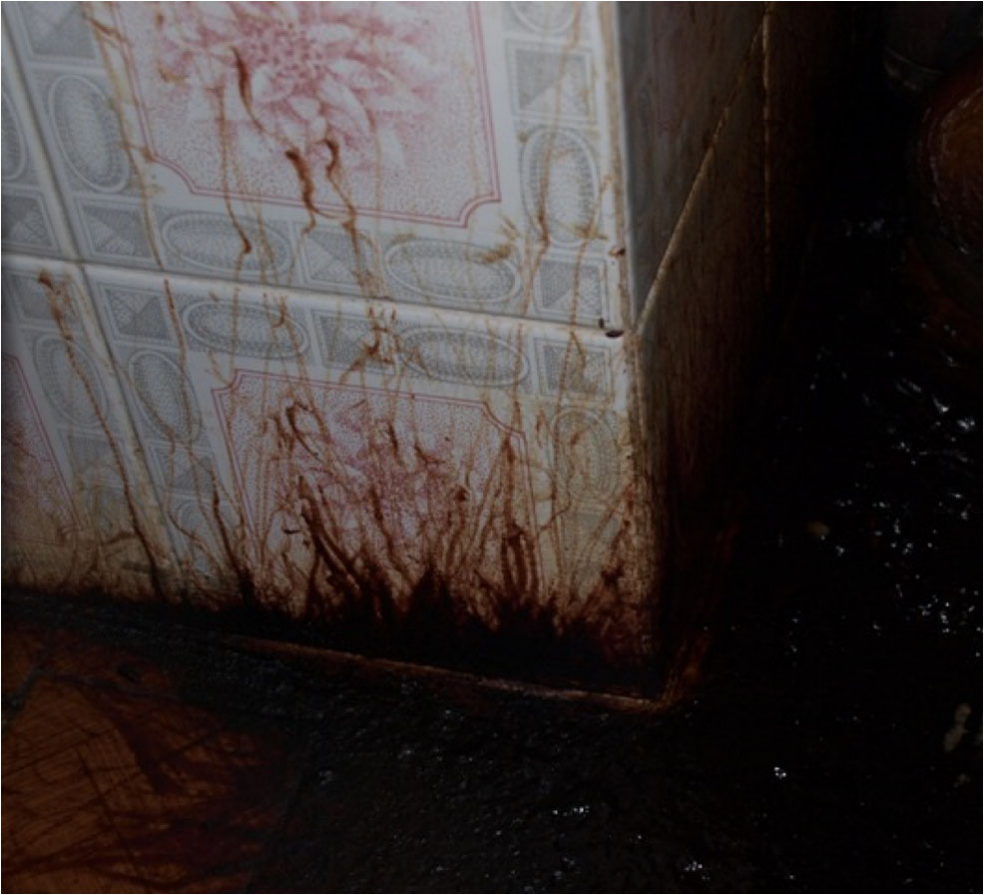
Stripes due to the maggots’ movement from the body can be present as well on vertical surfaces. Stripes generally show a large base and a thinner end resulting from the progressive release of blood or other decomposition fluids on the surface (Photo by *L. Bassi*)

Diptera larvae, called maggots, in the families Calliphoridae, Sarcophagidae and Muscidae are the primary consumers of animal organic matter. After completing their feeding phase, the majority of the larvae (e.g. *Calliphora* spp., *Lucilia* spp., etc) disperse to find an adequate place for pupariation and then pupation (the final developmental stage of metamorphosis into the adult stage) whereas a minority remain on the corpse or in the clothes (e.g. *Phormia regina* and *Protophormia terraenovae*). This phase of wandering larva is called “postfeeding” because at this stage, larvae stop feeding and empty the digestive system from any food remains [54] (Figs. 1 and 2). Larvae can migrate up to 10 m from the body, towards sheltered areas [8, 58]. During this process, if migrating from a surface soaked by blood or putrefactive liquid, maggots could leave a linear wipe pattern, resulting in a series of trails produced by their typical crawling motion. This pattern could be misinterpreted as an attempt to move the body, as a wipe pattern produced by third subjects or by the victim itself in the dynamic of the crime/death (e.g. swipe patterns created by bloody hair) (Fig. 1).

Not only fly maggots but also cockroaches, through the action of their tarsi, can produce blood-like droplets. They are larger than those produced by blowflies and consist in a series of tracks that often have a centre drag mark or “smear”, caused by the cockroaches dragging its abdomen [7]. The main differences with FAs are that patterns produced by blowflies are isolated, smaller and not as uniform [7]. Artefacts produced by cockroaches, due to their discontinuity, are also easily distinguishable from that produced by larvae.

### Entomological body artefacts

In forensic investigations, the estimation of the origin and the age of ante- and peri-mortem injuries is one of the fundamental steps to identify the cause of death [35] that, as previously mentioned, is one of the key points for crime reconstruction. Moreover, after death, corpses go through a series of transformations and alterations resulting from cellular and tissue lysis, from seaweed, fungi and plants proliferation and from local macro- and micro-fauna feeding activity [16, 39, 46]. In particular, faunas, both micro (e.g. insects) and macro (e.g. carnivores and other scavengers), play an important role in the flesh removal causing different kinds of macroscopic alterations of the body.

This kind of post-mortem alteration deserves the forensic pathologist’s attention when evaluating corpses either at the crime scene or during the autopsy [35]. In fact, the underestimation of such alterations may cause considerable complications in clarifying the cause of death, leading to wrong conclusions [59].

**Fig. 3:**
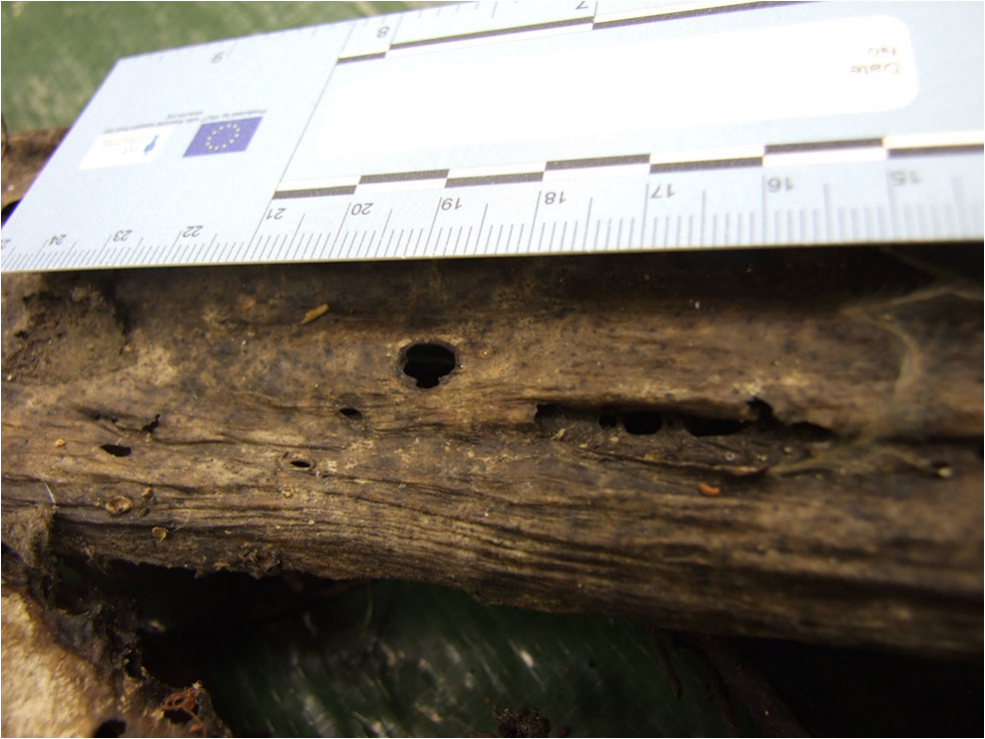
Circular skin lesion produced by maggots on the skin of a victim’s leg. Some damages of tissues may resemble to lesions inflicted ante-mortem (Photo by S. Vanin)

In particular, post-mortem damage of tissues may result in lesions that resemble inflicted or accidental ante-mortem injuries (Fig. 3). Furthermore, the post-mortem body alterations can cause modifications of the real ante-mortem wounds (i.e. pattern, size, shape), with loss of identifying features and damage or removal of internal organs [36].

The scavenging activity varies considerably depending on the animal feeding on the body with variation associated with the region, the season and the environmental conditions [16] and sometimes the distinction between ante- and post-mortem injuries is very difficult to be detected.

In particular, insect artefacts can take place on superficial ante-mortem injuries, resulting in the modification of wounds and/or loss of identifying features, for instance a gunshot wound or even more nail abrasions in the neck after manual strangulation [39].

Furthermore, injuries resulting from post-mortem arthropod activity can confuse even experienced pathologists in terms of the nature and chronology of the injury, due to similarities with the ante-mortem wounds so that they may be misidentified as sources of intravenous drug use, bite marks, defensive wounds, ulcers, burns or abrasions, signs of an attack, abuse, neglect or torture or other activities depending on the case and death scene circumstances [35, 38].

Regarding post-mortem arthropod activity, the alterations can be divided according to the affected tissues and, for each tissue, according to the type of arthropods involved (Table 2).

**Table 2:** Post-mortem alterations associated with arthropod activity

	Taxon	Injury description	Differential diagnosis
Skin and other soft tissue alterations	Ants	- Small punctate or multifocal areas of skin loss with small linear trails bordering the primary erosion points. - Irregular, serpiginous, scalloped areas of superficial skin loss known as “parchmenting”.	- Abrasions, cigarette or strong acids scars. - Patterned abrasion due to the imprinted effect of a blunt or offending object. - Marks from manual or ligature strangulation.
	Beetles	- Undulations, small pits, holes, grooves and tunnels in skin and connective tissues with bites on the edges of the wounds.	- Imprinted effect of a blunt or offending object. - Brush or chemical burns.
	Crustaceans	- Extensive but superficial wounds with haemorrhagic aspect, variable shape and vague irregular outlines, varying from 3 to 10 mm.	- Chemical or deep second-degree burns.
	Cockroaches	- Small (< 0.6 cm), round physical artefacts on the skin.	- Burns.
	Mecoptera	- Round lesions of the epidermis and part of the superficial dermis, approximately 3–5 mm in diameter, with an alopecic border. The centre of the lesion is brown–red and slightly eroded.	- Cigarette scars.
Bone alterations	Beetles, moths, wasps and termites	- Sub-parallel striations, edge gnawing, pits, holes, nests, tunnels and etching of the bone surface.	- Gunshot entrances.
Hair alterations	Beetles, moths	- Concave lesions caused by “gnawing” activity.	- Sharp force trauma.

### Skin and other soft tissue alterations

Among the arthropods, the most abundant species active in the body decomposition belong to the orders Diptera and Coleoptera [35, 36]. However, the arthropods described in the literature as causes of skin and soft tissues alterations that may be confused with ante-mortem injuries are ants (Hymenoptera, Formicidae), beetles (Coleoptera) and aquatic organism (e.g. Crustacea, Isopoda and Amphipoda) and the main literature on this topic concerns case reports [13–17, 20]. In addition, in the last year, a case of injuries on pig skin was reported from a forested area in Chile as caused by scorpion flies in the family Eomeropidae (Mecoptera) [60].

On the contrary, despite the large diffusion of cockroaches (Blattodea), especially in degraded and dirty environments, little information is reported about their activity on the bodies.

#### Ants

Ants are one of the world’s dominant insect groups with important ecological roles. Their part in the faunal succession varies from predator of eggs and larvae of other insects to early scavenger on the flesh or exudates from the corpse itself. Their activity was documented both in outdoor and indoor cases [45].

**Fig. 4:**
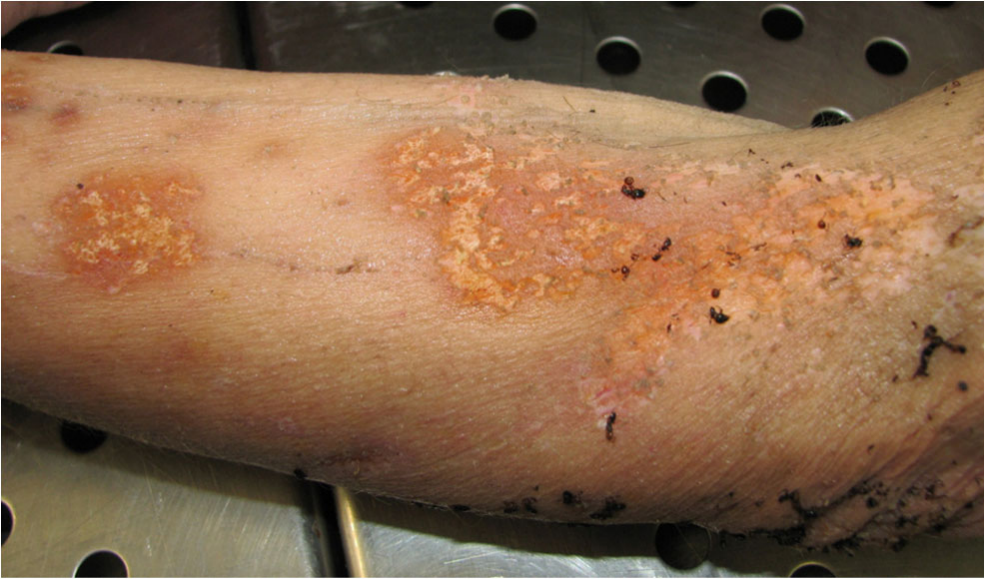
Irregular skin lesions of the arm produced by ant feeding activity on the cadaver. Ants can affect the interpretation of the tanatological data both removing fly larvae and producing post-mortem lesions orange-pink to yellow in colour diffusely scattered over the skin surface (Photo by *L. Bassi*)

On a body, ants feed off keratin in eyelashes, eyebrows, lips and knuckles and superficial layers of skin. Their feeding pattern is confined and characterised by multifocal areas of skin loss with small linear trails bordering the primary erosion points. The resultant thinning of the epidermis leads to an increased fluid loss with the development of irregular, serpiginous and scalloped areas of superficial skin loss known as “parchmenting” [36]. In addition, small punctate and scratch-type lesions may be observed on the body [45]. Usually, ant injuries are orange-pink to yellow in colour and diffusely scattered over the skin surface, with a quite alarming appearance [32, 45, 61] (Fig. 4).

Post-mortem injuries caused by Formicidae [35, 36] can be easily misinterpreted as ante-mortem lesions, such as abrasions, cigarette or strong acids scars, patterned abrasion due to the imprinted effect of a blunt or offending object and marks from manual or even ligature strangulation, especially when located on the neck and restricted by the collar line of a T-shirt or pullover.

As insects feed mainly on the uncovered areas of the body, ant bites are frequently located on the arms and the legs [20, 45]. The absence of bleeding was used to distinguish them from ante-mortem injuries. However, sometimes, considerable haemorrhage can take place, especially when removal of superficial layers of skin occurs in congested or hypostatic parts of the body [18].

Therefore, the final diagnosis can be only confirmed at the autopsy by gross and microscopic analysis.

Histologically, the level of skin damage inflicted by the feeding action of ants is often represented by the absence of the epidermis only (the top layer of the skin that is totally removed by ant bites exposing the dermis and underlying structures) without haemorrhage from the skin and the subcutaneous tissues.

Ant injuries may be of some utility in recording the presence of clothing and the position of the body after death since ant injuries can mark the perimeter of clothing and outline the junction between the body and the surface that it was laying on, due to their inability to gain access to skin beneath elasticized clothing or parts of the body pressed against the floor or ground. This information may be important especially if clothing has been removed prior to autopsy or if lividity is minimal [20].

#### Beetles

Skin (Dermestidae), chequered (Cleridae), clown (Histeridae) and burying (Silphidae) beetles can appear on a body during the late decomposition phases as body feeders or since the early stages as predators of other species. Beetles cause alterations that can be confused with lesions that could actually cause the victim’s death [40]. It has been observed that beetles may cause depressed areas and destruction on wounds, with bites on the edges of the wounds. Beetles’ bites may produce undulations, small pits, holes, grooves and tunnels in skin and connective tissues, generally starting from the skin, which can mimic ante-mortem injuries or can be easily misinterpreted as patterned abrasion due to the imprinted effect of a blunt or offending object or to brush or chemical burns [39].

#### Aquatic organisms

Cadavers collected from water often show post-mortem lesions caused by water turbulence, contact with the bottom of the river but as well as by the feeding activity of fluvial and marine scavengers, the latter including cookiecutter sharks, small size fish, deep sea crabs and mollusks [62–64].

Post-mortem injuries caused by aquatic animals [17] need to be differentiated from ante-mortem wounds [14] to obtain a clear understanding of the peri-mortal events but as well as to reconstruct the potential point of entrance of the body into the water.

The crustacean scavenging action is mentioned in many forensic textbooks but the aspect of these injuries is not described in detail in the literature, probably because of the difficulties in attributing the observed wounds to a specific species, with a few exceptions [15].

**Fig. 5:**
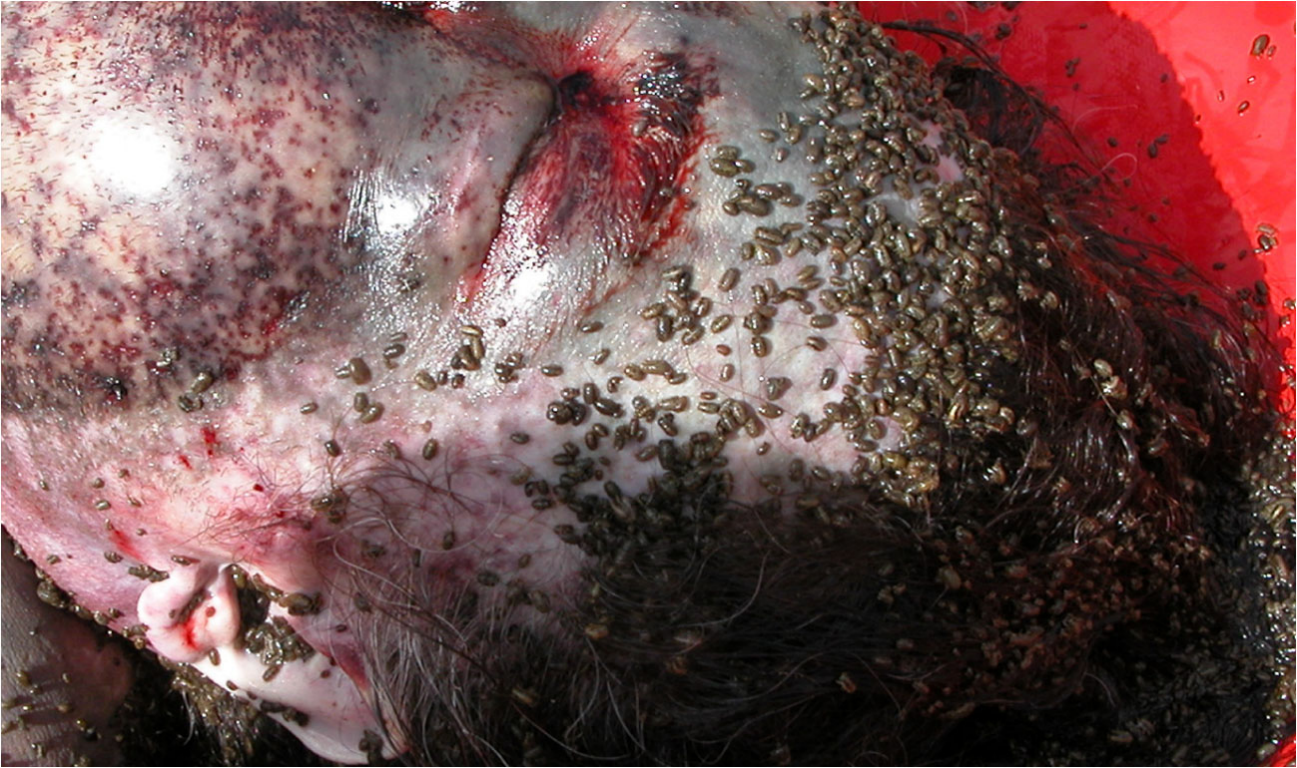
Post-mortem skin lesions caused by sea isopods feeding activity on the cadaver. The same pattern was reported as well for fresh water amphipods (e.g. *Niphargus* sp.) [8] (Photo by *L. Bassi*)

The post-mortem damage of the body in freshwater can include the loss of soft tissues mainly on the face and in cervical area. The haemorrhagic aspect of extensive but superficial wounds predominantly on the face can gave rise to suspicion of criminal assault (Fig. 5). After drying, the wounds around the eyelids present a roughly circular aspect, centred on the eyes with irregular outlines. The lips can be more profoundly damaged with losses of both mucous and muscular tissues. The skin of the neck, the back of the hands and of the abdomen just above the belt can have a similar aspect characterised by epidermal poorly haemorrhagic abrasion areas of variable shape, with vague irregular outlines, varying from 3 to 10 mm, with complete absence of vital reaction [13, 15].

Such a particular macroscopic aspect of these can to lead to the erroneous diagnosis of chemical or deep second-degree burns [14].

#### Cockroaches

Literature reports that cockroach feeding activity on remains can be misinterpreted as burn since they left small (< 0.6 cm), round physical artefacts on the skin [23].

#### Scorpionflies

Some round lesions of the epidermis and part of the superficial dermis, approximately 3–5 mm in diameter, with an alopecic border were reported on the skin of pigs used for decomposition studies in South America [60]. The centre of the lesion was brown–red and slightly eroded. The lesions that for the authors had morphological similarities with ante-mortem injuries caused by cigarette burns were performed by *Notiothauma reedi* (Mecoptera: Eomeropidae). No record of this kind of lesions were reported from human cases.

### Bone alterations

A variety of insect taxa can modify bones with their mandibles, including the larvae and adults of some beetles (i.e. Dermestidae, Tenebrionidae, Scarabaeoidea), moths (Tineidae), wasps (Halictidae, Sphaecidae) and termites (i.e. Termitidae, Mastotermitidae, Rhinotermitidae) [31], as observed upon human remains in archaeological contexts [40].

In particular, terrestrial invertebrates, which have an osteophagic behaviour, can colonise the burial place and influence the taphonomic processes [24] even in completely skeletonized bodies, with destruction of bones associated with different forms of star shaped features, clusters of microscopically visible sub-parallel striations, edge gnawing, pits, holes, nests, tunnels and etching of the bone surface [21, 33]. Such holes can be misinterpreted as gunshot entrances.

### Hair alterations

Hairs are the most resistant structures in the human body after teeth and bones and nails and can be collected from a crime scene several years after a victim’s death. However, they can be altered by different organisms, such as fungi and arthopods, mainly insects in the orders Coleoptera and Lepidoptera. A general classification of damage has indicated that insect feeding activity only produces concave lesions caused by their “gnawing” activity, whereas fungal alteration leads to transversal tunnelling inside the hair structure. Studies of trauma on hair are extremely rare but a pilot study highlighted that the effect of the entomological components involved seems to be clearly distinguishable (thanks to the “gnawing” pattern) from sharp force trauma [44].

## Procedural notes proposal

The crime scene, as well as the body, which can be considered itself as a crime scene, must be analysed and scrutinised with attention and precision, also taking in consideration the possibility of the post-mortem alterations due to the activity of arthropods and other organisms (e.g. mammals, birds, fungi).

On this point, the analysis of the literature has shown that among the 15 papers dealing with the issue of post-mortem arthropod body injuries, histological analysis were performed only in three cases in order to confirm post-mortem nature of the injuries while in the remaining 12 papers, the macroscopic findings were not microscopically confirmed.

Therefore, in order to reduce the risk of misinterpretation, some procedural notes to be followed in case of potential alterations due to the insect activity are suggested here. These notes take into account the two phases of a violent crime investigation: the crime scene investigation and the autopsy, and they can be applied also in a broader context when other animals or environmental factors could affect the integrity of the body.

### Crime scene investigation

**Fig. 6:**
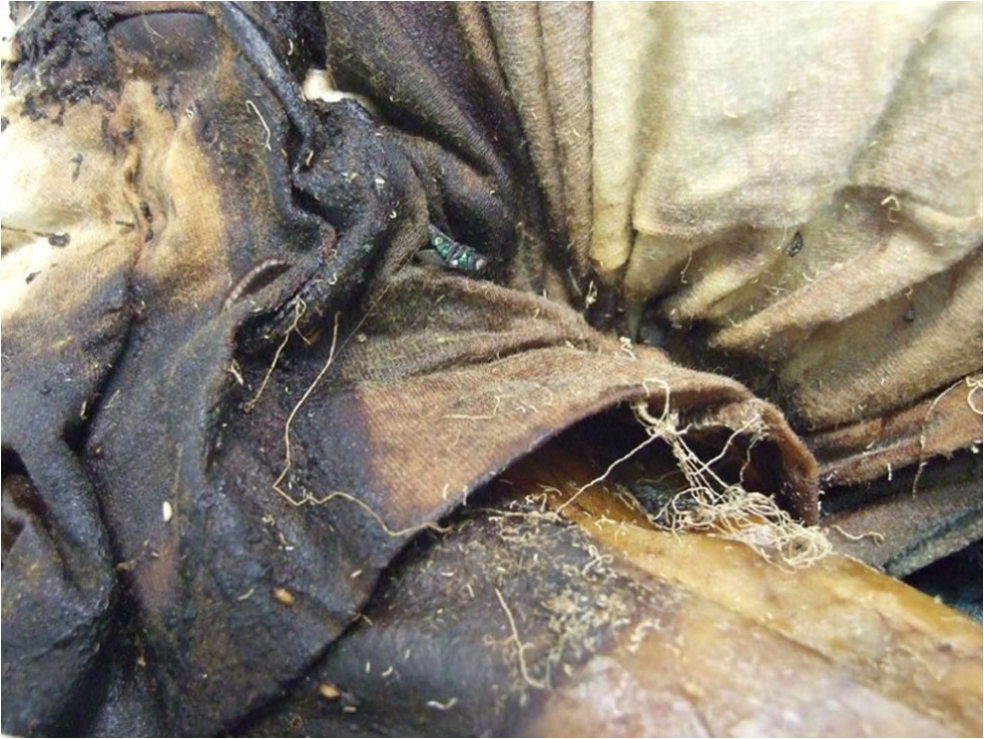
Larder beetle (Coleoptera, Dermestidae) frass on a leg of a partially mummified body that appears as whitish ribbons (photo by *S. Vanin*)

- Perform complete and detailed description and photographic documentation with a proper camera of the crime scene, including body position, clothes position, potential FAs and floor stripes position that may be present, at the discovery moment.
- Pictures have to be collected using the principles of forensic photography, since commercial smartphones and tablet do not have the appropriate resolution. All the detail photos have to be done with and without an appropriate scale and an optimization of the light.
- Windows, doors and lights positions have to be reported on the documentation in order to identify any entry/exit point for insects and other animals. In indoor cases, particular attention has also to be paid not only to the room where the body lays but as well to the contiguous spaces, under the carpet or any object on the floor potentially used as a shelter by the insects.
- Collect all the arthropods present on or near the body or in the crime scene [3, 38] and then store them in ethanol (70–100%) and sent to a specialist for identification. As an alternative, insects can be frozen and later prepared as requested by the specialists. Furthermore, in the absence of living arthropods, the presence of faecal pellets, frass and exuviae has to be documented since they could be used as an indicator of the previous arthropods’ presence [40], and in case of frass, it can also be used as a substratum for toxicological analyses (Fig. 6). If insects have to be used for minimum post-mortem estimation, DNA or drug analysis refer to the specific guidelines [5].

### Autopsy

- Search and collect insects present on the body, clothes, body bag or coffin after transport from the crime scene to the morgue.
- Perform an accurate external examination on clothes and on the body, describing and photographing all the injuries with and without an appropriate scale and an optimization of the light.
- Compare the position in which the body was found and the clothes with the site of injuries.
- Perform an incision into each lesion area, and underlying tissues should be observed and sampled for further histological analyses.
- In case of suspicion of lesions being produced by arthropods, a histological analysis of the samples has to be performed, in order to confirm the post-mortem nature of the lesions, characterised by the absence of vital reaction, such as congestion, fibrin deposit or inflammatory reaction.

## Conclusions

Entomological artefacts represent an important issue for forensic pathologists, since they can lead to wrong investigative reconstruction and misinterpretations, both during body examination at the crime scene and during the autopsy.

Therefore, in order to avoid misinterpretations and evaluation errors in the discrimination between ante-mortem injuries and post-mortem insect artefacts, the correct training in forensic pathology should include expertise regarding post-mortem artefacts produced by arthropods and other organisms. Moreover, during the different phases of a violent crime investigation (i.e. crime investigation and autopsy), the potential existence of alterations due to insect activity, animals or environmental factors that can affect the integrity of the body must be considered in order to take the necessary operational and procedural attentions, for example, following the tips in Table 2.

In conclusion, a multi-disciplinary approach is recommended in death investigation [32, 44, 65], and when insect activity is detected, a close co-operation between the forensic pathologists and forensic entomologists is highly encouraged, in order to provide an high quality death investigation and more accurate and precise evaluation.
